# Ultra-low-dose chest CT protocol during the second wave of COVID-19 pandemic: a double-observer prospective study on 250 patients to evaluate its detection accuracy 

**DOI:** 10.1186/s43055-021-00512-2

**Published:** 2021-05-26

**Authors:** Ahmed Samir, Reham Mohamed El-Husseiny, Rania Ahmed Sweed, Nermeen Abd El-Monsef Abd El-Maaboud, Mohamed Masoud

**Affiliations:** 1 Department of Radiodiagnosis and Intervention, Faculty of Medicine, University of Alexandria, Alexandria, Egypt; 2 Ministry of Health and Population, Cairo, Egypt; 3 Department of Chest Diseases, Faculty of Medicine, Alexandria University, Alexandria, Egypt

**Keywords:** COVID-19, Low dose, Ultra-low dose, HRCT

## Abstract

**Background:**

While the second wave of COVID-19 pandemic almost reached its climax, unfortunately, new viral strains are rapidly spreading, and numbers of infected young adults are rising. Consequently, chest high-resolution computed tomography (HRCT) demands are increasing, regarding patients’ screening, initial evaluation and follow up. This study aims to evaluate the detection accuracy of ultra-low-dose chest CT in comparison with the routine low-dose chest CT to reduce the irradiation exposure hazards.

**Results:**

This study was prospectively conducted on 250 patients during the period from 15th December 2020 to 10th February 2021. All of the included patients were clinically suspected of COVID-19 infection. All patients were subjected to routine low-dose (45 mAs) and ultra-low-dose (22 mAs) chest CT examinations. Finally, all patients had confirmatory PCR swab tests and other dedicated laboratory tests. They included 149 males and 101 females (59.6%:40.4%). Their age ranged from 16 to 84 years (mean age 50 ± 34 SD). Patients were divided according to body weight; 104 patients were less than 80 kg, and 146 patients were more than 80 kg. HRCT findings were examined by two expert consultant radiologists independently, and data analysis was performed by other two expert specialist and consultant radiologists. The inter-observer agreement (IOA) was excellent (96–100%). The ultra-low-dose chest CT reached 93.53–96.84% sensitivity and 90.38–93.84% accuracy. The signal-to-noise ratio (SNR) is 12.8:16.1; CTDIvol (mGy) = 1.1 ± 0.3, DLP (mGy cm) = 42.2 ± 7.9, mean effective dose (mSv/mGy cm) = 0.59 and absolute cancer risk = 0.02 × 10^-4^.

**Conclusion:**

Ultra-low-dose HRCT can be reliably used during the second wave of COVID-19 pandemic to reduce the irradiation exposure hazards.

## Background

Real-time polymerase chain reaction (PCR) remains the gold standard tool for the diagnosis of the Novel coronavirus disease (COVID-19) since it was first described in December 2019 and announced as a pandemic in February 2020 [1]. However, in mild cases with low viral load, many patients had false-negative swab tests [2]. The test also remains time-consuming, and PCR kits had not increased enough to face the increased number of patients all over the world [3, 4].

On the other hand, high-resolution computed tomography (HRCT) expressed more availability and more rapid results. Besides, HRCT sensitivity exceeded 90% in comparison with PCR sensitivity which could not exceed 75% [5]. Consequently, most of the publication since then recommended routine non-contrast HRCT of the chest for the evaluation of COVID-19 [6–8]. Low-dose CT was then introduced to decrease the ionizing irradiation risk of exposure [9].

In September 2019, the American Association of Physicists in Medicine (AAPM) suggested certain criteria for efficient low-dose CT during lung cancer screening among standard-sized persons (70–90 kg), including CTDIvol ≤ 3.0 mGy, DLP ≤ 75 mGy cm and effective dose ≤ 1.0 mSv [10]. A dose of 50 mAs was suggested before in 2016 for low-dose CT screening of intrathoracic abnormalities [11].

While the second wave of COVID-19 pandemic almost reached its climax, unfortunately, new viral strains are rapidly spreading, and numbers of infected young adults are rapidly rising. Hence, chest HRCT demands are increasing.

This study aims to evaluate the detection accuracy of ultra-low-dose chest CT in comparison with the routine low-dose chest CT during the assessment of COVID-19 patients in order to reduce the irradiation exposure hazards.

## Methods

### Study population and design

This study was prospectively conducted on 250 patients who were clinically suspected of COVID-19 infection during the period from 15th December 2020 to 10th February 2021. The study was approved by the Ethics Committee of our University hospital. Patient verbal consent was accepted by the Ethics Committee respecting absolute safety of non-invasive non-therapeutic procedure without additional personal risk or burden to the public health, also assuring full respect of both patient and medical record confidentiality. The authors emphasize on the fact that the overall radiation dose in both low- and ultra-low-dose CT examinations together remains much lower than single routine chest CT examination.

Patients included 149 males and 101 females (59.6%:40.4%). Their age ranged from 16 to 84 years (mean age 50 ± 34 SD). By definition of the American Association of Physicists in Medicine (AAPM), the standard bodyweight for an adult ranges from 70 to 90 kg [10]. Hence, patients were divided according to body weight: 104 patients were less than 80 kg, and 146 patients were more than 80 kg (Mean weight 84.4 ± 11.1 SD). This would eventually help to determine the accuracy of the suggested CT dose in relation to the variable body weight. The maximum weight reached in this study without image distortion was 107 kg.

All patients were initially subjected to routine low-dose CT protocol (45 mAs) [11], then all patients were examined by a new suggested ultra-low-dose CT examination (22 mAs). Finally, all patients had confirmatory PCR swab tests and other dedicated laboratory tests including; CBC, CRP, D-dimer, serum ferritin, LDH, procalcitonin, and sputum culture. Clinical and laboratory evaluation was performed by a single expert consultant pulmonologist.

*Inclusion criteria were as follow:* patients with clinical history of suspected COVID-19 infection including acute onset of either fever, cough, chest pain, dyspnea, body aches, sore throat, loss of smell sensation or loss of taste sensation.

*Exclusion criteria were as follow:* (1) Poor quality of CT images because of patients’ tachypnea and motion artefacts and (2) non-available PCR test results or inconclusive final diagnosis.

### CT scanning (machines and protocols)

Non-contrast CT chest examinations were performed in a single radiology center using a single multi-detector computed tomography machine: Aquilion 16, Canon Medical system Toshiba, Tustin, CA, USA. The scanning parameters and the CT dosing measurements of both low-dose and ultra-low-dose CT protocols exactly followed the guidelines of AAPM [10]. They are detailed in (Table 1) in correlation to the body weight. Adaptive Iterative Dose Reduction (AIDR) was used in all examinations to reduce the irradiation dose while maintaining an acceptable image quality. Smooth images were obtained at 40%.

**Table 1:** Scanning parameters for the initial “low-dose” and suggested “ultra-low-dose” CT protocol according to body weight

	CT protocol			
	Initial “low dose”		Suggested “ultra-low dose”	
	Body weight			
	< 80 kg	≥ 80 kg	< 80 kg	≥ 80 kg
**Scanning parameters**				
**kV**	120	120	120	120
**mA**	60	60	30	30
**Rotation time**	0.75	0.75	0.75	0.75
**mAs (mA × time)**	45	45	22	22
**Pitch**	1.4	1.4	1.4	1.4
**Eff mAs (mAs/pitch)**	32	32	16	16
**Slice thickness (mm)**	5	5	5	5
**Slice thickness recon**	1.5	1.5	1.5	1.5
**Field of view (FOV)**	350	350	350	350
**Matrix size**	512 × 512	512 × 512	512 × 512	512 × 512

### CT scanning (radiation dose calculation and cancer risk estimation)

The effective dose in millisievert (mSv) was calculated according to the American Association of Physicists in Medicine by multiplying the total dose length product (DLP) by conversion coefficient (0.014 mSv/mGy cm) [12]. The mean risk for developing cancer in populations who were exposed to irradiation (absolute cancer risk) is calculated by multiplying the forementioned effective dose by the risk coefficient (0.041 Sv^−1^) [13].

### CT scanning (data evaluation)

The HRCT findings of the low-dose and ultra-low-dose CT examinations were assessed among two groups of patients (A: less than 80 kg and B: more than 80 kg) by two expert consultant radiologists independently, and data analysis was performed by other two expert radiologists (their experience ranged 6–14 years). They were informed of the patients’ clinical history.

*The universal HRCT features of COVID-19 were respected which include* ground-glass opacities (GGOs), solid nodules with GG halo, consolidative changes, fibro-atelectatic changes and sub-pleural curvilinear bands, crazy-paving pattern (GG admixed with septal thickening) and tree in bud nodules.

The HRCT image evaluation was performed using multi-planar reconstruction (axial, sagittal and coronal planes). The minimum intensity projection (Min-IP) reconstruction was used for adequate evaluation of the ground glass attenuation. The maximum intensity projection (MIP) reconstruction was used for evaluation of the septal thickening, crazy paving pattern, and tree in bud nodules.

*The minimum attenuation/density of the pathological region*s was measured in both low-dose and ultra-low-dose CT examinations among both groups of patients using the Hounsfield unit (Hu). This would eventually help to detect not only the accuracy of the ultra-low-dose CT protocol but also the percentage of error regarding the lung attenuation.

**Fig. 1:**
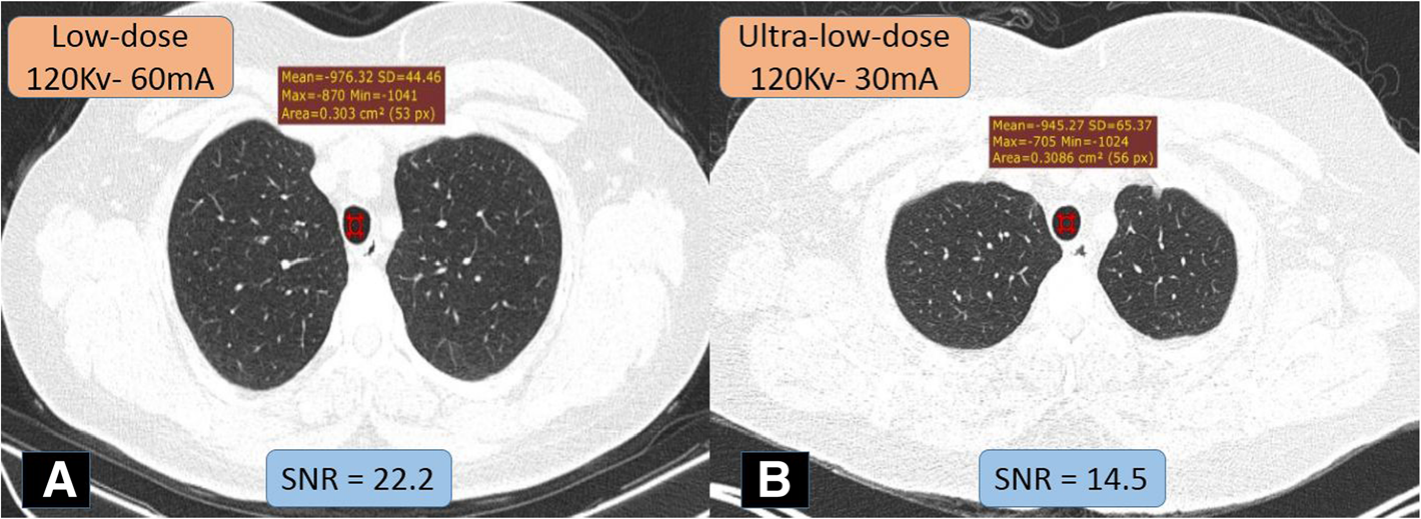
Image quality at highest and lowest SNR; measured at axial chest CT cuts/lung window (− 700:1500) within the tracheal lumen immediately above the aortic arch at standardized 0.3 cm^2^ ROI (SNR = mean attenuation/SD). **a** Low-dose CT with higher SNR in a 77-kg patient and **b** ultra-low-dose CT with lower SNR in a 85-kg patient; there is no significant blurring at lung image; however, mild blurring is noted at the shadow of the thoracic cage muscles

*The image quality* was assessed objectively using the signal-to-noise ratio (SNR). It is calculated through the following formula [SNR = Density of air / SD of air] [14]. The standard deviation (SD) of the region of interest (ROI) is measured within the tracheal lumen just above the level of the aortic arch. It is calculated three times in the lung window setting (window level = − 700 HU; window width = 1500 HU), then the mean value is estimated. Certain ROI surface area is unified (0.3 cm^2^) [15]. By definition, the higher the signal-to-noise ratio, the better the resolution (Fig. 1) [16].

### Statistical analysis

The prevalence rate of each HRCT finding (isolated or mixed) was estimated as the percentage of patients with each abnormal CT finding. This was done by each observer for both groups of patients regarding both low-dose and ultra-low-dose CT examinations.

Similarly, the prevalence rate of the maximum attenuation of the pathological lesions was also estimated.

Cohen’s Kappa test was utilized to calculate the “Inter-Observer Agreement” (IOA) coefficient regarding each HRCT characteristic and lung attenuation.

An online calculator (https://www.medcalc.org/calc/diagnostic_test.php) was utilized to detect the mean value and 95% confidence interval (95% CI) regarding the sensitivity, specificity, positive predictive value (PPV), negative predictive value, positive likelihood ratio, negative likelihood ratio, and accuracy of both low-dose and ultra-low-dose CT protocols.

## Results

### CT dose calculations and cancer risk estimation (Table 2)

The main differentiating parameter between the routine low-dose CT protocol which was previously implemented for screening of lung cancer and the new ultra-low-dose CT which is suggested for assessment of COVD-19 pneumonia was the intensity of the irradiation. At the low-dose CT, mA was 60, mAs was 45 and Eff mAs was 32. In ultra-low-dose CT, the mA was 30, mAs was 22 and Eff mAs was 16. Other parameters were similar. Consequently, there was a decrease in the CT dose measurements. CTDIvol dropped from 1.6 ± 0.4 mGy to 1.1 ± 0.3 mGy. DLP dropped from 60.9 ± 9.5 to 42.2 ± 7.9 mGy cm. The mean effective dose also dropped from 0.85 to 0.59 mSv/mGy cm. Absolute cancer risk consequently dropped from 0.03 × 10^-4^ to 0.02 × 10^-4^.

**Table 2:** CT dosing measurements and image quality for the initial “low-dose” and suggested “ultra-low dose” CT protocols according to body weight

	CT protocol			
	Initial “low dose”		Suggested “ultra-low dose”	
	Body weight			
	< 80 kg	≥ 80 kg	< 80 kg	≥ 80 kg
**CT dose**				
**CTDIvol (mGy)**	1.6 ± 0.4	1.6 ± 0.4	1.1 ± 0.3	1.1 ± 0.3
**DLP (mGy cm)**	60.9 ± 9.5	60.9 ± 9.5	42.2 ± 7.9	42.2 ± 7.9
**Mean effective dose (mSv/mGy cm)**	0.85	0.85	0.59	0.59
**Absolute cancer risk**	0.03 × 10^-4^	0.03 × 10^-4^	0.02 × 10^-4^	0.02 × 10^-4^
**Image quality**				
**Objective image noise (SD)**	39.1–44.4	48.3–54.1	59.5–61	65.9–73.8
**Signal-to-noise ratio (SNR)**	21.2–24.4	17.5–19.8	15.3–16.1	12.8–14.5

### Image quality (objective SNR) (Table 2)

The image quality was minimally decreased in the ultra-low-dose CT protocol. The signal-to-noise ratio (SNR) in the ultra-low-dose CT was lower than that in the low-dose CT (12.8–16.1 compared with 17.5–24.4 respectively). This almost did not impact the diagnostic efficacy of CT as detailed later on.

### Prevalence of HRCT findings (Table 3)

Ground-glass opacities were the most common findings among our patients. They were found alone in 173 patients (69.2%). They were found also surrounding a solid nodule (ground-glass halo sign) among 49 patients (19.6%) and associated with interlobular septal thickening (crazy paving pattern) also among 49 patients (19.6%). Consolidative changes with or without ground-glass opacities were detected among 84 patients (33.6%). The CT attenuation of these pathological CT findings ranged from + 40 to − 750 HU. The predominant CT attenuation among these CT findings ranged from – 300 to − 400 HU (157 patients/62.8%).

**Table 3:**
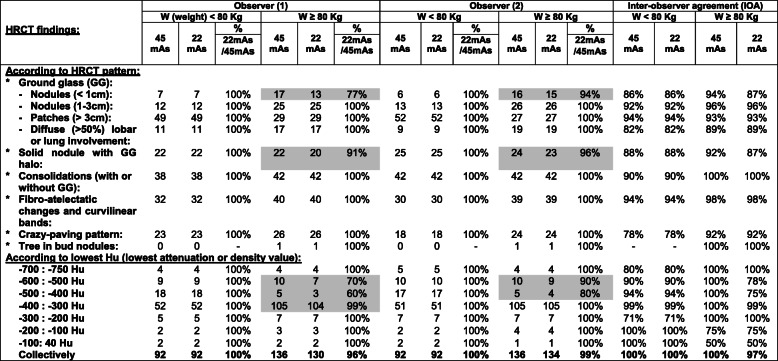
Distribution of HRCT findings (CT patterns and CT density) at low dose (45 mAs) and ultra-low dose (22 mAs) among 228 patients with more or less than 80-kg body weight, performed by two separate observers

### Detection ability of HRCT findings of COVID-19 and their CT attenuation (Table 3)

#### Among 104 patients with < 80-kg body weight

**Fig. 2:**
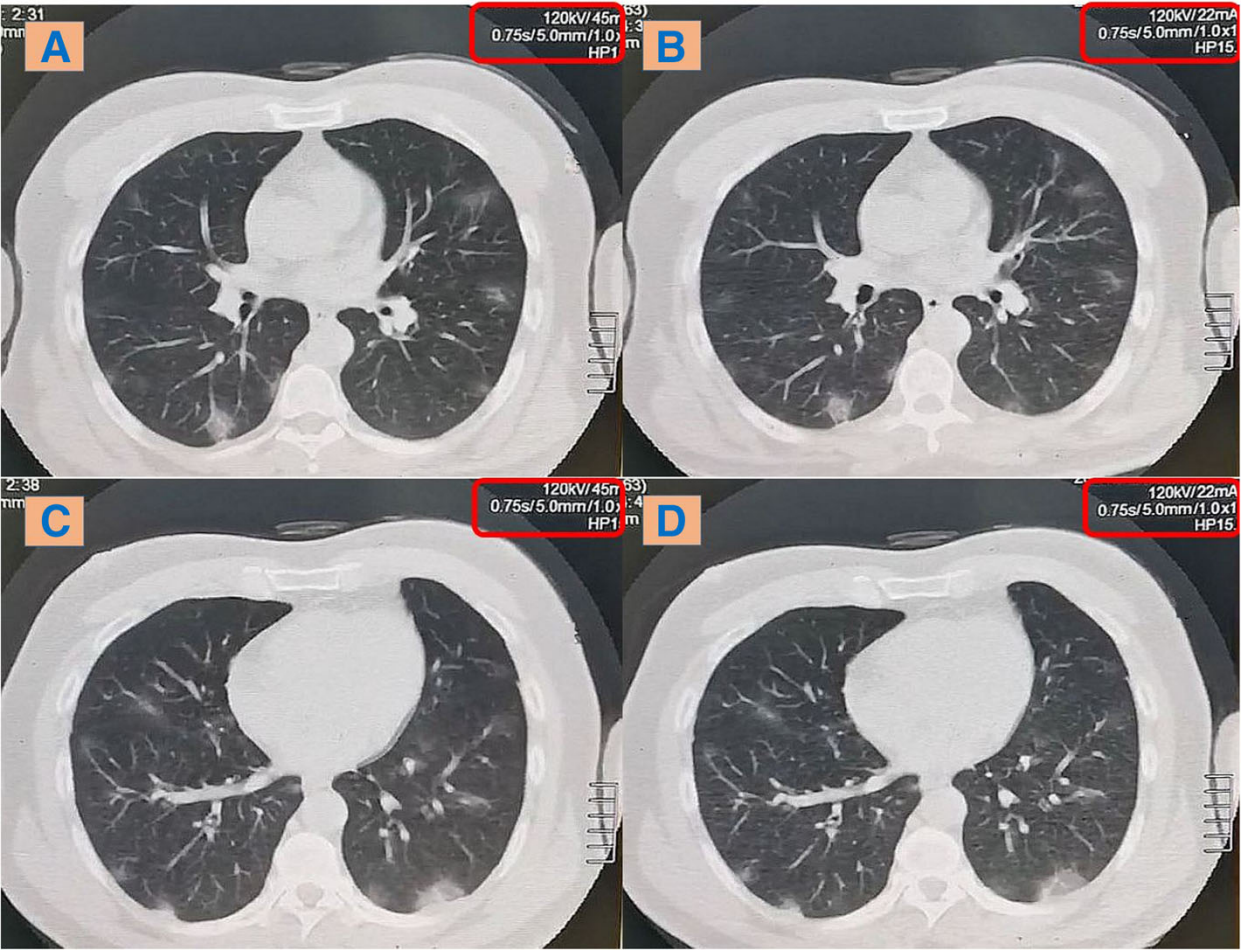
A 72-kg male patient complaining from anosmia and sore throat; axial CT chest lung window showing bilateral sub-pleural scattered ground-glass patches. **a** & **c** Low-dose CT. **b** & **d** Ultra-low-dose CT

**Fig. 3:**
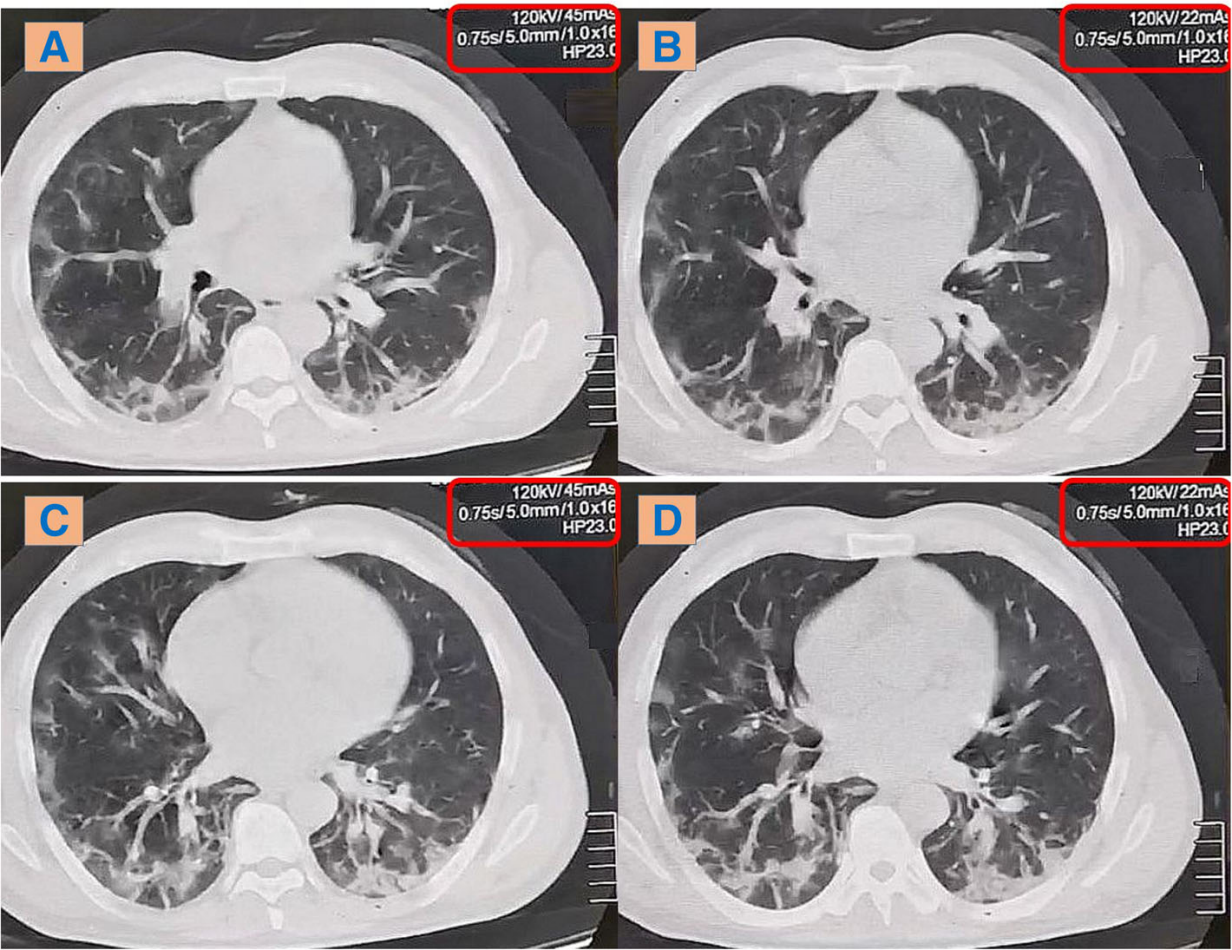
A 75-kg male patient complaining from fever, cough and dyspnea; axial CT chest lung window showing bilateral sub-pleural scattered ground-glass patches with fibro-atelectatic changes. **a** & **c** Low-dose CT. **b** & **d** Ultra-low-dose CT

In comparison with the low-dose CT, the ultra-low-dose CT protocol similarly detected all HRCT findings of COVID-19 (including the ground-glass opacities, consolidations, fibrotic changes, Atoll sign and crazy paving pattern), as well as their ranges of CT attenuation (+ 40 to − 750 HU). The inter-observer agreement was excellent (100%) (Figs. 2 and 3).

#### Among 146 patients with ≥ 80-kg body weight

**Fig. 4:**
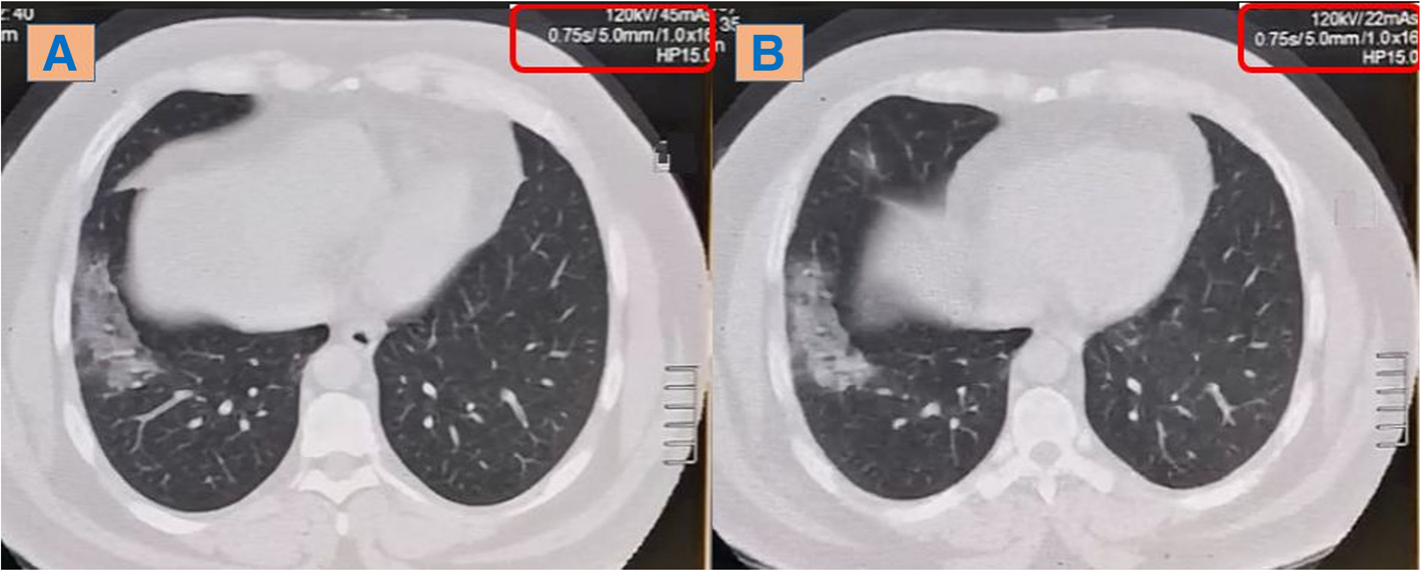
A 83-kg male patient complaining from fever and cough; axial CT chest lung window showing right lower lobar single ground-glass patch. **a** Low-dose CT. **b** Ultra-low-dose CT

**Fig. 5:**
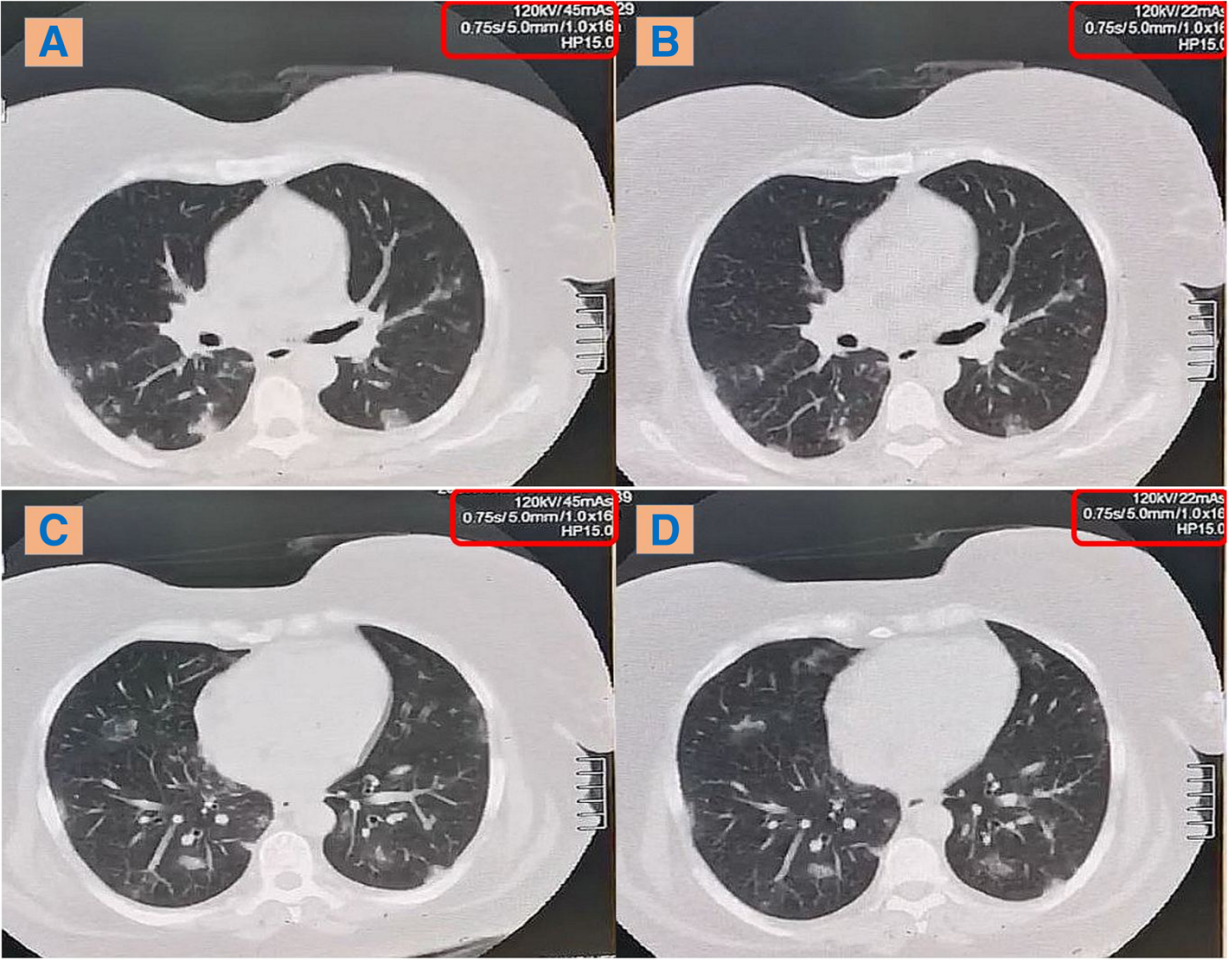
A 95-kg female patient complaining from fever and dyspnea; axial CT chest lung window showing bilateral sub-pleural scattered ground-glass patches. **a** & **c** Low-dose CT. **b** & **d** Ultra-low-dose CT

**Fig. 6:**
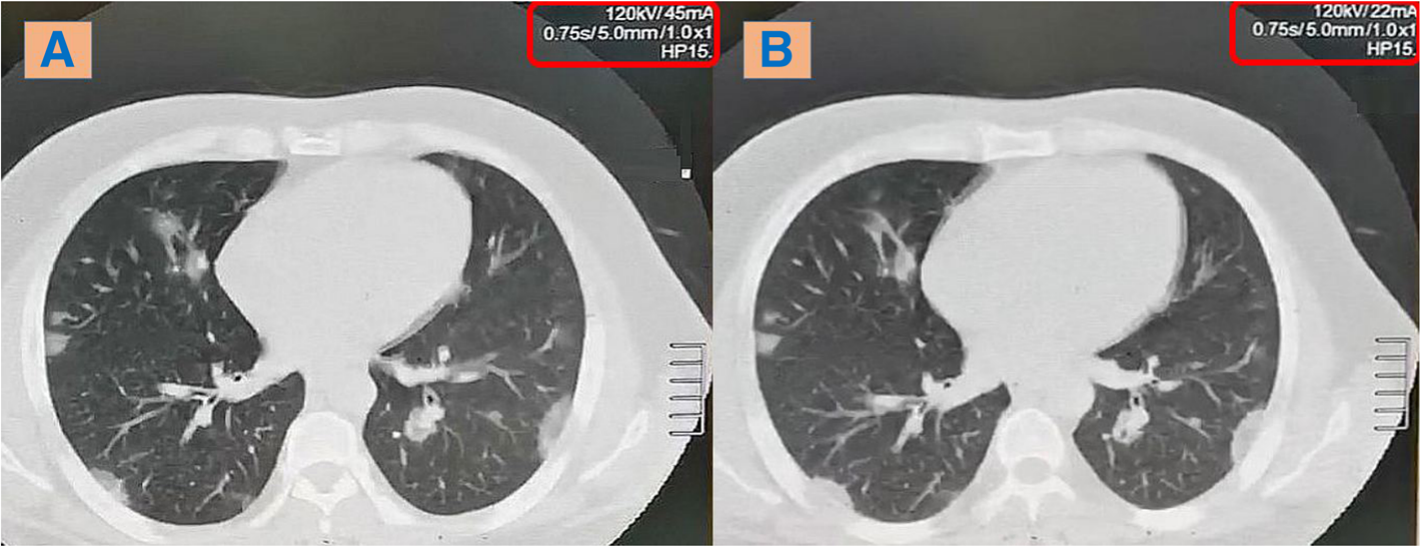
An 85-kg male patient complaining from fever and cough; axial CT chest lung window showing bilateral sub-pleural scattered ground-glass patches. **a** Low-dose CT. **b** Ultra-low-dose CT

In comparison with the low-dose CT, the ultra-low-dose CT protocol only showed lower efficacy regarding detection of small GG nodules < 1 cm and nodules with ground-glass halo sign (ranging from 77 to 94% and 91 to 96% among both observers respectively). The CT attenuation of these lesions ranged from − 300 to − 600 HU. Otherwise, no absolute differences were found regarding the detection of the rest of the HRCT findings and their ranges of CT attenuation. Finally, the inter-observer agreement was collectively excellent (96–100%) (Figs. 4, 5 and 6).

### Final diagnosis and statistical differences between low-dose and ultra-low-dose CT

**Fig. 7:**
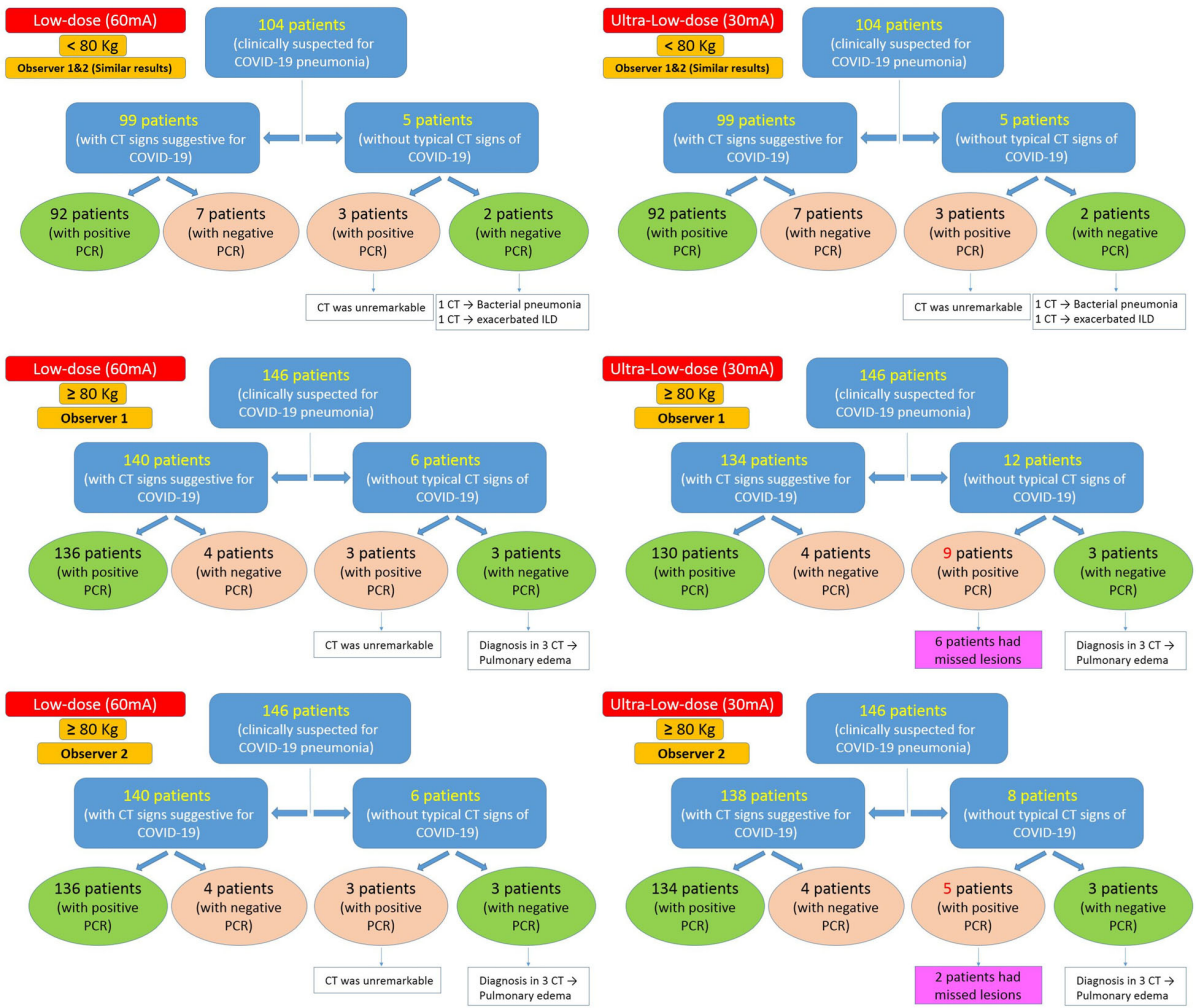
Multi-compartmental flow chart summarizing the CT results for both observers and final diagnoses after PCR results, regarding low-dose and ultra-low-dose CT examination at patients less than and more than 80-kg body weight

A multi-compartmental flow chart (Fig. 7) is summarizing the CT results for both observers and final diagnoses after PCR results; regarding low-dose and ultra-low-dose CT examination for patients less than and more than 80-kg body weight.

Collectively the ultra-low-dose chest CT reached 93.53–96.84% sensitivity and 90.38–93.84% accuracy in comparison with the low-dose CT which showed 96.84–97.84% sensitivity and 90.38–95.21% accuracy.

#### Among 104 patients with < 80-kg body weight (Table 4)

No differences were found between both observers at both low-dose and ultra-low-dose CT protocols regarding the final diagnosis. Ninety-two patients (88.5%) were truly positive and confirmed by PCR to have COVID-19. On the other hand, only 3 patients (3%) were false negative. Consequently, both CT protocols showed same statistical results: sensitivity (mean = 96.84% and CI 95% = 91.05 to 99.34%) and accuracy (mean = 90.38% and CI 95% = 83.03 to 95.29%).

**Table 4:** Summary of statistical results for “low-dose” and “ultra-low-dose” CT protocols in patients (< 80-kg body weight)

Statistical results	Low-dose CT (45 mAs)				Ultra-low-dose CT (22 mAs)			
	Observer 1		Observer 2		Observer 1		Observer 2	
	Value	95% CI^**a**^	Value	95% CI	Value	95% CI	Value	95% CI
**Sensitivity**	96.84%	91.05 to 99.34%	96.84%	91.05 to 99.34%	96.84%	91.05 to 99.34%	96.84%	91.05 to 99.34%
**Specificity**	22.22%	2.81 to 60.01%	22.22%	2.81 to 60.01%	22.22%	2.81 to 60.01%	22.22%	2.81 to 60.01%
**Positive predictive value (PPV)**	92.93%	90.25 to 94.92%	92.93%	90.25 to 94.92%	92.93%	90.25 to 94.92%	92.93%	90.25 to 94.92%
**Negative predictive value (NPV)**	40.00%	11.31 to 77.70%	40.00%	11.31 to 77.70%	40.00%	11.31 to 77.70%	40.00%	11.31 to 77.70%
**Positive likelihood ratio**	1.25	0.88 to 1.77	1.25	0.88 to 1.77	1.25	0.88 to 1.77	1.25	0.88 to 1.77
**Negative likelihood ratio**	0.14	0.03 to 0.74	0.14	0.03 to 0.74	0.14	0.03 to 0.74	0.14	0.03 to 0.74
**Accuracy**	**90.38%**	83.03 to 95.29%	**90.38%**	83.03 to 95.29%	**90.38%**	83.03 to 95.29%	**90.38%**	83.03 to 95.29%

^a^*CI* confidence interval

CT specificity was equally low (22.22%) for both low-dose and ultra-low-dose CT as 7/99 patients showed false-positive CT results with negative PCR swab tests and alternative diagnoses (two patients had fungal pneumonia, and five patients had aspiration pneumonia).

#### Among 146 patients with ≥ 80-kg body weight (Table 5)

Observer 1 truly detected 130 patients using ultra-low-dose CT compared with 136 patients using low-dose CT. Six cases were furtherly missed using the ultra-lose-dose CT. Consequently the low-dose CT showed sensitivity (mean = 97.84% and CI 95% = 93.82 to 99.55%) and accuracy (mean = 95.21% and CI 95% = 90.37 to 98.05%), while the ultra-low-dose CT showed sensitivity (mean = 93.53% and CI 95% = 88.06 to 97.00%) and accuracy (mean = 91.10% and CI 95% = 85.26 to 95.17%).

**Table 5:** Summary of statistical results for “low-dose” and “ultra-low-dose” CT protocols in patients (≥ 80-kg body weight)

Statistical results:	Low-dose CT (45 mAs)				Ultra-low-dose CT (22 mAs)			
	Observer 1		Observer 2		Observer 1		Observer 2	
	Value	95% CI^**a**^	Value	95% CI	Value	95% CI	Value	95% CI
**Sensitivity**	97.84%	93.82 to 99.55%	97.84%	93.82 to 99.55%	93.53%	88.06 to 97.00%	96.40%	91.81 to 98.82%
**Specificity**	42.86%	9.90 to 81.59%	42.86%	9.90 to 81.59%	42.86%	9.90 to 81.59%	42.86%	9.90 to 81.59%
**Positive predictive value (PPV)**	97.14%	94.71 to 98.48%	97.14%	94.71 to 98.48%	97.01%	94.47 to 98.41%	97.10%	94.63 to 98.45%
**Negative predictive value (NPV)**	50.00%	19.64 to 80.36%	50.00%	19.64 to 80.36%	25.00%	10.32 to 49.12%	37.50%	15.13 to 66.88%
**Positive likelihood ratio**	1.71	0.90 to 3.25	1.71	0.90 to 3.25	1.64	0.86 to 3.11	1.69	0.89 to 3.21
**Negative likelihood ratio**	0.05	0.01 to 0.21	0.05	0.01 to 0.21	0.15	0.05 to 0.44	0.08	0.02 to 0.28
**Accuracy**	**95.21%**	90.37 to 98.05%	**95.21%**	90.37 to 98.05%	**91.10%**	85.26 to 95.17%	**93.84%**	88.62 to 97.14%

^a^*CI* confidence interval

Meanwhile, observer 2 truly detected 134 patients using ultra-low-dose CT compared with 136 patients using low-dose CT, while only two cases were furtherly missed using the ultra-low-dose CT. Consequently the low-dose CT showed sensitivity (mean = 97.84% and CI 95% = 93.82 to 99.55%) and accuracy (mean = 95.21% and CI 95% = 90.37 to 98.05%), while the ultra-low-dose CT showed sensitivity (mean = 96.40% and CI 95% = 91.81 to 98.82%) and accuracy (mean= 93.84% and CI 95%= 88.62 to 97.14%).

CT specificity was equally low (42.86%) for both low-dose and ultra-low-dose CT as 4/146 patients showed false-positive CT results with negative PCR swab tests and alternative diagnoses (two patients had exacerbated interstitial pneumonia, and two patients had aspiration pneumonia).

Comparison with other CT protocols is demonstrated in Table 6, regarding patient groups, dose parameters and final CT protocol results.

**Table 6:** Comparison between “low-dose” and “ultra-low-dose” CT protocols in this study and previous “low-dose” CT protocols

	Previous studies				Current study	
	Tabatabaei et al. [17]	Dangis et al. [18]	Bahrami-Motlagh et al. [19]	Shiri et al. [20]	Routine low dose	Suggested new ultra-low dose
**Number of patients**	20	192	163	-	250	250
**Age (mean)**	-	67 years	-	-	50 years	50 years
**Weight**	-	-	-	-	84 kg	84 kg
**kV**	120	100	110–120	90	120	120
**mA**	30	20	20–30	20–45	60	30
**Pitch**	1	1.2	1.4–1.5	0.8	1.4	1.4
**Rotation time**	-	0.5	0.6–0.75	-	0.75	0.75
**Slice thickness**	3	-	-	-	5	5
**Slice thickness recon**	-	1	-	-	1.5	1.5
**FOV**	-	450	-	-	350	350
**IOA**	98–99%	92–98%	-	-	100%	97–100%
**CTDIvol (mGy)**	3.5	1.3	1.8	0.7	1.6 ± 0.4	1.1 ± 0.3
**DLP (mGy.cm)**	112	40	65	-	60.9 ± 9.5	42.2 ± 7.9
**Mean effective dose (mSv/mGy cm)**	1.8	0.56	0.9	-	0.85	0.59
**Absolute cancer risk**	0.74 × 10^-4^	0.02 × 10^-4^	0.04 × 10^-4^	-	0.03 × 10^-4^	0.02 × 10^-4^
**Accuracy**	-	-	**96.6%**	-	**95.21%**	**91.10–93.84%**

## Discussion

The second wave and even further crisis of COVID-19 pandemic are striking the world with increased demands for chest CT imaging [21].

Similar to Chung et al. [22] and Song et al. [23], pure ground-glass opacities were the most common HRCT finding of COVID-19 in this study. According to Xia et al. [17], a solitary pure GG nodule could be the first sign of COVID-19 infection. This eventually increased the challenge during the lowering of the CT dose.

According to Kubo et al. [11], the DLP was 764.3 mGy cm, the mean effective dose was 10.7 mSv/mGy cm, and absolute cancer risk was 0.4 × 10^-4^. These estimated radiation doses are 12.5:13.3 and 18.1:20 times more than that of the low-dose and the ultra-low-dose CT protocols in this study respectively. Additionally, the DLP and absolute cancer risk of the ultra-low-dose CT in this study were lower than those provided by Tabatabaei et al. [18], Dangis et al. [19] and Bahrami-Motlagh et al. [20]. On the other hand, the CTDIvol was higher than that provided by Shiri [24] (0.7 mGy) who used lower kV (90 kV) and lower pitch (0.8).

The inter-observer agreement in this study was excellent, similar to Tabatabaei et al. [18] and Dangis et al. [19]. The accuracy in this study was also high but did not exceed that of Bahrami-Motlagh et al. (96.6%) [20]. The CT sensitivity also in this study exceeded that in Azadbakht et al. [25] (60–70%).

Comparison with Tabatabaei et al. [18], Dangis et al. [19], Bahrami-Motlagh et al. [20] and Shiri [24] regarding the CT protocol parameters as well as the estimated irradiation doses and CT accuracy is demonstrated in Table 6.

The quality of CT images in this study was generally accepted and did not impact the accuracy of CT diagnosis. The SNR of the ultra-low-dose CT in this study even approximated that of the low-dose CT in Kang et al. [6].

The merits of this study over the previous literature were the following: the higher number of included patients compared with previous researches; also the estimation of effective dose, cancer risk, sensitivity and accuracy of the ultra-low-dose CT among two groups of patients with low and high body weight using double-observer assessment; and finally the analysis of the CT attenuation in addition to the HRCT findings to determine those with better performance and those with an acceptable percentage of errors.

The study was limited by a short time interval since the beginning of the second wave of the COVID-19 pandemic. The authors encourage further future studies on higher-slice machines to compare the results using filtered back projection (FBP) and iterative dose reduction (IDR).

## Conclusion

Ultra-low-dose HRCT can be reliably used during the second wave of COVID-19 pandemic to reduce irradiation exposure hazards.

## Data Availability

The datasets used and/or analyzed during the current study are available from the corresponding author on reasonable request.

## Abbreviations

COVID 19: Coronavirus disease 2019
GGOs: Ground-glass opacities
HRCT: High-resolution computed tomography
RT-PCR: Reverse transcription–polymerase chain reaction
DLP: Dose length product
AAPM: American Association of Physicists in Medicine
